# Report of the National Vaccine Establishment

**Published:** 1813-08

**Authors:** Charles Murray

**Affiliations:** Secretary. Sun Court, Cornhill


*To the Editors of the Medical and Physical Journal.*

GENTLEMEN,

I AM desired by the Board of the National Vaccine Establishment to request that you will insert in your next publication the enclosed Report of the Board printed by order of the House of Commons, in order that the same may be as extensively known as possible.

I am, Gentlemen,

Your most obedient Servant,

*Sun Court, Cornhill,* CHARLES MURRAY,

*July* 14, 1813. Secretary.

REPORT *of the* NATIONAL VACCINE ESTABLISHMENT,
*Dated April* 22, 1813.

*To the Right Hon. Viscount Sidmouth, Principal Secretary of State, Home Department, &c. &c. &c.*

*National Vaccine Establishment,*
MY LORD, *Leicester-square, April* 22, 1813.

The Board of the National Vaccine Establishment have the honor of informing your Lordship, that during the year 1812, the surgeons appointed by their authority to the nine stations in London, have vaccinated 4521 persons, and have distributed 23,219 charges of vaccine lymph to the public. The number vaccinated this year exceeds that of 1811 by 1373, and the demand for lymph has been often so great that it could not without difficulty be supplied. The Board had last year reason to think that nearly two-thirds of the children born in the metropolis, were vaccinated by charitable institutions or private practitioners. There is now reason to believe that three-fourths of those born are submitted to that salutary operation. But though the prejudices against the cow pock, which have been artfully encouraged by ignorant and interested men, appear generally to decline in the metropolis, as well as in other parts of these dominions, yet it is with concern that the Board have noticed the increase of mortality from small-pox in this city last year, to the number of 1287.

Previous to the discovery of vaccination, the average number of deaths from small-pox, within the bills of mortality, was 2000; and, though in the last ten years 133,139 persons were added to the population of this great city, yet in 1811, by the benefit of vaccination, the mortality was reduced to 751. The increase in the last year we have reason to ascribe to the rash and inconsiderate manner in which great numbers are still inoculated for the small-pox, and afterwards required to attend two or three times a-week, at the place of inoculation, in every stage of their illness. This practice of inoculation, and of promiscuous intercourse of the patients at the same time with society, is the great means by which this disease is kept in existence, and its infection propagated to persons and places where it would not otherwise be seen. This is not only the opinion of this Board, founded on observation, but it is a fact confirmed by communications to them from the best authorities, and by the most unprejudiced characters.

The respectable College of Surgeons of Dublin allege that the practice of inoculation not only supplies a constant source of infection, but prevents the extinction of the disease, for even a short interval.

The populous city of Norwich was never free from it till the discovery of vaccination, but since that period it has experienced occasional remissions from its ravages. In 1807, after its disappearance for some time, the disorder was brought into that city by a vagrant from London, who, before the magistrates were apprised of it, or before the salutary advice given by the faculty to provide a place where such person might be secluded from intercourse with the inhabitants could be adopted, communicated the contagion. Of 1200 who took the infection, 203 died. At that period, viz. 1807, the prejudices against vaccination had not subsided. But in 1812, when that city was threatened with a similar visitation, by the appearance of the small-pox in the neighbourhood, the magistrates, the faculty, and the clergy, concurred in recommending vaccination. Between the 10th of August, and 22d of October following, 1316 persons were vaccinated. The result was, that though one gentleman, whose child the faculty refused to inoculate, procured matter of small-pox, which he applied himself, and from this source, seven persons took the infection, yet by means of this seasonable vaccination not a life was lost.

This result, so different from the events of 1807, cannot but make an impression on every mind open to conviction: when vaccination was not performed, 1200 persons took the small-pox, of which number 203 died; when speedy recourse was was had to vaccination, there was not a single victim to the disease.

But it is not at home only that lessons, so much to the credit of this new art, may be learned. The Board have abundant communications from every quarter of the world equally to its advantage. To detail all the evidence which they may have received as to its efficacy, not only in preventing the small-pox, but its power to suppress its ravages under the most unfavorable and threatening circumstances, would extend this Report to an improper and an unusual length. They will content themselves with mentioning a few particulars, which they trust will recommend it to the favor and confidence of their countrymen, and to the fostering care of government.

On the continent of India, vaccination has been hailed as the greatest blessing, and has been practised with the greatest success, and in the most extensive manner.

In the islands of Ceylon and Bourbon it has been received in a manner no less favorable, and been practised with an effect no less beneficial. In the isle of Ceylon, since its first introduction, more than 200,000 persons have been vaccinated; 30,491 in the year 1811 only, as appears by the account from Mr. Anderson, the Superintendant General, to whom but one case of failure, in preventing the small-pox, (and the circumstances of this case render it very doubtful) has occurred, in the great numbers which he has seen.

At the Cape of Good Hope the small-pox is dreaded as much as the plague, and it has proved there little less destructive to human life. Lord Caledon, the late governor, established at Cape Town a Vaccine Institution, which was soon called into activity under his successor Sir J. Cradock. The colony consists of a population of 80 or 100,000 individuals, of which number it was supposed 15,000 were subject to take the infection of the small-pox, which appeared there on the 12th of March, 1812. Between that time and the 4th of July following, 233 persons caught the disease, of which number 100 died. The remaining part of the inhabitants liable to the disorder were preserved by an active vaccination, in which all the faculty in the place, as well as the regimental and garrison surgeons, strenuously exerted themselves.

From the various details with which the Board have been favored, we think it our duty to select one instance, as tending to show in a most pointed manner the power of the vaccine lymph to arrest the contagion of the small-pox.

Four hundred negroes from Mosambique were, on the 1st of March, landed at Cape Town, one of whom, a woman, was on the 5th succeeding afflicted with the confluent small-pox in its most virulent form. This female was at that time inhabiting a large room in common with 200 more of her companions, not separated either by day or by night. On the report of this case, the whole of these victims of "avarice and cupidity," as the surgeon terms them, were immediately subjected to vaccination, and on the following day removed to a small island (Paarden Island) at a little distance from the town. A few days after this the woman fell a sacrifice to the most aggravated character of that dreadful disease. Of the aggregate number of negroes, 78 individuals received the vaccine disorder, and underwent the regular course of its action. From these subjects the remaining portion were vaccinated. "They remained on the island fifty days, during which no further case of small-pox made its appearance, although they had been exposed to the whole strength of the contagious atmosphere; nor is there a single instance wherein any of this large proportion of persons became subject to the small-pox." It is added by the professional gentleman who writes this account, that throughout the entire course of this "arduous struggle" (the general vaccination) not a single instance had come to his knowledge of the failure of vaccination in protecting the individual from the small-pox, where the former was ascertained to have taken effect.

At the Havannah, by the account written by Dr. Thomas Romey, Secretary to the Committee of Vaccination, 13,447 persons were vaccinated in 1810; 9315 of these persons had been vaccinated in the City of Havannah alone, with so good an effect, that for two years not a single person had been interred in the public burying ground of that city who died of the small-pox, which before was a great cause of mortality in it.

In the Caraccas, and in Spanish America, the small-pox has been extinguished by vaccination. For the means which were taken by the Spanish government, and its subjects, we must refer to the subjoined papers, furnished by some Spanish gentlemen now in London.

The accounts from various parts of Europe are almost as favorable. In the Report of last year it was observed, that the small-pox was extinguished at Milan, and at Vienna, in which latter place for many years the average mortality from it had amounted to 800.

From Malta information has been received that not only his Majesty's ships are supplied with lymph to vaccinate such such sailors as may not have had the small-pox, but, that the children of the artificers of the dock-yard, and nearly 3000 Maltese children, have been vaccinated by the Institution there (gratis): and it is added by Mr. Allen, the surgeon of the Dock-yard, that during a residence of seven years at Malta, he has never known an instance of one of them being afterwards afflicted with the small-pox.

Russia has likewise participated in the benefit of vaccination. It was introduced into the Russian empire in 1804; and since that time, in its various provinces, 1,235,637 have been vaccinated; and so uniformly successful has vaccination been, that it has been termed, in the language of that country, the *pock of surety*. Dr. Crighton, Physician to the Emperor of all the Russias, observes, supposing (according to a well-founded rule of calculation) that before the introduction of vaccination every seventh child died annually of the small-pox, vaccination has saved the lives, in the Russian Empire, of 176,519 children, since the year 1804.

The government of France appears to have taken the greatest pains to secure to the people all the advantages which could be derived from this discovery. A central institution was soon established at Paris, to encourage and to promote the practice of vaccination, and a similar plan for the same purpose was adopted in every considerable provincial town. These provincial institutions were not long ago ordered to make a return to the government of the state of vaccination in their several districts. From these documents a Report has been drawn up by M. Berthollet, Percé, and Halle, philosophers of the first reputation, and submitted to the class of Physical Sciences of the Imperial Institute; in which it is affirmed, that of 2,671,662 subjects, properly vaccinated in France, only seven cases appear of patients having afterwards taken the small-pox; which is as 1 to 381,666. It is added, that the well-authenticated instances of persons taking the small-pox after inoculation for that disease had perfectly succeeded, are proportionably far more numerous; and also that in Geneva, Rouen, and several other large cities, where the Jennerian system has not been circumscribed by popular prejudice, the small-pox is no longer known; and the Registers exhibit strong evidence of consequent increasing population. The Report concludes with expressing great hopes that this pestilential disorder will ultimately disappear from society.

This object will doubtless be greatly forwarded by the line of conduct adopted by the Royal College of Surgeons in London, in which city, notwithstanding the artifices practised, and the falsehoods* even propagated to discredit vaccination, it is even now gaining ground. The Royal College of Surgeons have resolved not to inoculate with variolous matter. The College of Surgeons of Dublin have formed the same resolution. In Gloucestershire sixty-three surgeons, convinced of the pernicious tendency of inoculation to support and propagate the small-pox, associated, and pledged themselves to decline the practice of it.

The National Vaccine Establishment have recommended the imitation of such examples to the members of the profession in every part of these dominions, and they have no doubt but that the good effects of such advice will soon appear, in the diminished mortality and the increased population of the country.

It may be proper to add, that the surgeons at nine stations of this metropolis, reported to us on the 14th of last January that they had no complaint of any person vaccinated by them having afterwards had the small-pox.

The Board have again the pleasure of stating, that the money granted by Parliament during the last session has been sufficient to defray the expenses of the year 1812; and they are of opinion that the same sum will be adequate to the expenditure of the current year.

FRANCIS MILMAN, President,

*By Order of the Board,*

JAMES HERVEY, M.D. Register.

---

EXTRACTS *from the* APPENDIX.

*Copy of a Letter from the President of the Royal College of Physicians, Edinburgh.*

SIR, *February 20th,* 1813.

In reply to your letter of the 5th January, I am directed by the Royal College of Physicians to inform you, that during the year 1812 vaccination has continued to be practised in this city as formerly, with uninterrupted success; that there have been very few instances where inoculation for the small-pox has been insisted on; and that the mortality from natural small-pox has, in as far as the Royal Col-

---

* In the bills of mortality for the last year, the death of two persons was said to have been occasioned by the cow pock, but, upon investigation by the Board of the National Vaccine Establishment, they were found to have died from other causes, and the assertion was proved to be without foundation.

lege lege can judge, been very inconsiderable in this part of Scotland. I have the honor to be, Sir,

Your most obedient humble Servant,
JAMES HAMILTON, jun. M.A. President.

*To Dr. Hervey, Register of the National Vaccine Institution.*

---

*Communication from the Royal College of Surgeons of Edinburgh.*

The Royal College of Surgeons of Edinburgh, in reply to the request of the National Vaccine Board, have only to announce, as on former occasions, their unanimous and undiminished confidence in the security which vaccination affords against the small-pox. They have also every reason to believe that the public confidence remains undiminished. Among the higher ranks, vaccination continues to be universally practised, and though among the lower orders it has rather diminished for the last two or three years, the College attribute this entirely to the absence of any alarm from small-pox, and in no degree to a want of confidence in vaccination; for such want of confidence would naturally have led to applications for variolous inoculation; and this has not occurred within the knowledge of any member of the College.

The College regret that from the want of regular public registers they are unable to give any account of the mortality from small-pox in Scotland, or the proportion of the population that has been secured against small-pox by vaccination. They beg leave to suggest the propriety and importance of adopting some plan by which this knowledge may be obtained; for there is every reason to believe that as small-pox becomes more rare, vaccination will, among the lower orders, be still more neglected.

JAMES LAW, President.

*Edinburgh*, 15*th January*, 1813.

---

*Copy of a Letter from the President of the Faculty of Physicians and Surgeons in Glasgow.*

SIR, *Glasgow*, 17*th February*, 1813.

Your letter of the 5th of January having been laid before the Faculty of Physicians and Surgeons, a Committee was appointed to report thereon, and reported as follows:

"The Committee appointed to report to the Board of the National Vaccine Establishment, on the progress of vaccination in Glasgow, beg leave to state, that the deaths by small-pox in the year 1812, have in that city amounted to 24; whereas whereas the average number of deaths from 1801 to 1804 exceeded 100, and the deaths for the seven years previous to the introduction of vaccination exceed 200 yearly, though the population has of late years greatly increased; that eleven hundred and sixty-two have been gratuitously vaccinated at the Faculty Hall this year, besides the private patients of all the medical practitioners in town; and that the practice of inoculation for small-pox is totally discontinued, and the confidence in the preventive power of vaccination continues unabated."

(Signed) James Monteath.
B. W. King.
William Anderson.

The Faculty unanimously approve of this Report, and ordered a copy of it to be transmitted by the Præses to the Board of the National Vaccine Establishment.

I have the honor to be, Sir,
Your most obedient Servant,
J. Balmanno, M.D. Præses of Faculty.

---

### *Report of the Royal College of Surgeons in Ireland.*

Sir, *Dublin, February 5th*, 1813.

I have the honor to acknowledge the receipt of your letter of the 5th ultimo, addressed to the President of the Royal College of Surgeons in Ireland, requesting the further opinion of the College on the practice of vaccination and its effects; and inquiring if the practice of inoculation for the small-pox obtains in Ireland; and what may be the mortality from the natural small-pox during the year 1812: and I am directed by the College to state in reply thereto, that since they had the honor of communicating with you on this subject early in the last year, no circumstance has occurred to induce them to alter the favorable opinion then expressed on the practice of vaccination.

Genuine cow-pox, considered as a disease, appears to the College to be characterized by mildness, seldom induces any very obvious constitutional indisposition during its progress; and, it is believed, has uniformly proved an effectual prevention of small-pox.

A few cases of small-pox succeeding to vaccination have been reported to the College to have occurred since the last communication; but in these, either the cow-pox vesicle was imperfectly formed, or the other appearances, the existence of which is necessary to mark the true disease, were unsatisfactory. And further, the number of these cases is so small in proportion to that of vaccinated persons who are known to have have resisted variolous contagion, particularly during the year 1812, that the confidence hitherto placed by the College in the anti-variolous effects of cow-pox remains unshaken.

For several years the members and licentiates of the College of Surgeons, and it is believed, all regular physicians and apothecaries in Ireland, have adopted the practice of vaccination; but it has been ascertained that some unauthorized practitioners continue to inoculate for the small-pox, and thus renovate and support sources of contagion.

To this practice has been ascribed the prevalence of natural small-pox, as an epidemic, in Dublin, and throughout the country, during the greater part of last year; the mortality occasioned by which, the College regret to be obliged to state, was very considerable, but the number cannot be ascertained, as returns are not made by the parishes.

I have the honor to be, Sir,
Your most obedient Servant,
J. Henthorn, Sec.

*D. Hervey, M.D.*

*&c. &c. &c.*

---

*Translation of a Statement on the Vaccine Disorder, by Dr. Servando de Meir y Noriega, an Ecclesiastic.*

*Dated London, 10th Jan.* 1813.

The small-pox, as well as the measles, were unknown in New Spain before the conquest. They were brought there, says Torquemado,* by a Negro from Pamfilo of Narvaez, and they occasioned such destruction, that he does not hesitate to affirm, that the greatest part of the Indians died, among whom was the Emperor Cuitlahuatzin, who succeeded Montezume. It is stated, that according to the reports, which Cortes ordered to be made to him, there died in the empire of Mexico alone three millions and a half. It was not long before fresh variolous infection was brought over, and according to Torquemada eight hundred thousand Indians perished.

Europe has continued to communicate this scourge at intervals of thirty, twenty, or a less number of years, and the infection extending itself from Vera Cruz to the most remote parts, has like a destructive plague spread terror, death, and desolation, over that continent. The longer it is retarded, the more fatal it becomes, because the danger increases with the age of the sufferers. Thirty-three years ago there were carried off more than ten thousand persons in the towns of Mexico and Puebla alone by this contagion, which was the last but one that has visited that kingdom, and was brought

* A Spanish historian.

there after an interval of nineteen years. It was from this last attack that I was a sufferer in my native country, Monterry, the capital of the new kingdom of Leon: and there was not a family who did not put on mourning. Some of these families disappeared altogether, because they were all adult persons, and had been seized by the epidemic in the city. Those who lived in the country were preserved from its influence by banking the dung-hills of the large and small cattle around their dwellings.

The small-pox acts with the greatest virulence upon those parts of the body most exposed to the sun, such as the face and hands; and as the Indians are more exposed by their habit of life and manner of clothing, the havoc which it makes among them is more horrible.

Torquemada says, speaking of the first introduction of the infection, that the reason why it killed so many, was, because the Indians were ignorant of the nature of the disease, and bathed and scratched themselves.

In the new kingdom of Leon there were several wandering nations, so warlike that the Spaniards could not with arms in their hands resist their attacks upon their towns; the small-pox, however, extirpated almost all of them; and fifty years ago heaps of bones, like so many trophies of the disease, were to be seen under the old tufted oaks in the fields. At this present time, when a savage sees one of his companions attacked with the infection, he leaves him, his horse, and his provisions, and flies to a great distance in the woods.

It has never happened that the Spaniards have secured themselves against infection by stopping their communications with the Indians.

As soon as the inoculation for the natural small-pox was introduced into Europe, the Archbishop of Mexico, Haro, ordered the curates and ecclesiastics to perform it through their several towns with their own hands; and although the prejudices and scruples of some hindered the practice becoming general, it is certain that to this inoculation is to be attributed the diminished evil which the small-pox occasioned fourteen years ago.

The King of Spain having sent the art of vaccination with Dr. Balmis, it was received with such pompous ceremonies, both civil and military, that the people caught the enthusiasm. I believe that not a person remained at that time unvaccinated. The viceroy's lady herself, Dona Jues de Toregui, employed herself in vaccinating the Indian children. And as the vaccine is found in the cows in the provinces of Puebla and Michauacan, every body having it at hand, all the children are now vaccinated, and the small-pox has not appeared appeared for fourteen years. They already believe their country to be free from such a scourge, and should its contagion appear again in Vera Cruz, it would be easy to counteract it in the beginning by employing the vaccine, although its use might have been for some time laid aside.

The celebrated Dr. Unamie also writes at Luna, that in the two towns of the Sierra of Peru there had been no small-pox, because the inhabitants inoculated themselves by milking the cows who actually had the vaccine. Upon being asked, whether they had ever the small-pox, they answered, they only had a few pimples on their hands.

(Signed) Dr. SERVANDO DE MEIR Y NORIEGA.

---

*Translation from the Spanish.*

Having been secretary to the Junta, established in Caraccas, for extending the use of the variolous vaccine, I am enabled to authenticate the following facts. In the year 1803 the Spanish government fitted out an expedition for the purpose of transmitting to the Spanish establishments in America and Asia, this inestimable antidote against one of the most fatal scourges that has afflicted mankind, and which in the Spanish colonies of America has been particularly destructive, Dr. A. Francisco Xavier Balmis, private physician to the king, was appointed chief of the expedition, and to his care, and that of others of the faculty, were entrusted a number of children, sufficient to preserve the invaluable germ, communicated from arm to arm. One of the first places at which the expedition touched was the Caraccas, where the small-pox was reviving every spring, and committing no small ravages during that and the summer season. Inoculation had been long known in the Caraccas: however, this practice, indisputably beneficial to those individuals who employed it, was most fatal to the people at large, the majority of whom, either from superstition, or want of the means, could not enjoy its benefits ; so that the higher classes, recurring constantly to inoculation, contributed to perpetuate and extend the contagion, of which the people were the victims.

The nature of the colonial government of America afforded the Spanish government particular advantages towards the establishment, and the universal propagation of the variolous vaccine. Thus it was, that at the expiration of a few months after the arrival of the expedition, the small-pox was entirely exterminated in the department of Penezuela. The authority of the government, the influence of the clergy, and especially the experience of its salutary effects, together with the mildness of the operation, concurring, it was soon made general, and the children of every class were brought to the house established for the purpose, under the inspection of the Junta, to which I was some time secretary.

As the institution of this Junta was to watch over the effects of vaccination, for which purpose they communicated with the faculty of physic, and the curates of all the parishes in the department, I was enabled to ascertain, with the greatest certainty, that the success of this establishment has been in the Caraccas the most complete that can be imagined; and that only on some parts of the coast, where the population was so thin that they could not keep up yearly the vaccine fluid, the common small-pox has appeared twice. It, however, only attacked those who had not received its antidote. Equally good effects have been attested in the other parts of Spanish America, and, thanks to the illustrious Jenner, the population of this part of the world yearly receives an augmentation of 1,000,000 of lives, which, but for his glorious discovery, had fallen a prey to the small-pox.

One of the objects to which the Juntas employed in this branch have devoted their attention, was to promote investigation of the cow-pox in those districts in their respective provinces, where large herds of cattle are kept; and in the district of Calabozo, belonging to that of the Caraccas, they have had the satisfaction of finding it in the cows. The effects produced by the cow-pox originating in Calaboza, were entirely of the same nature with that brought from Europe, only it was observed that the irritation was something greater when they administered the indigenous fluid.

(Signed) A. BELLO.

*London, Jan.* 11, 1813.

